# The clinicopathological significance and prognostic value of programmed death-ligand 1 in prostate cancer: a meta-analysis of 3133 patients

**DOI:** 10.18632/aging.202248

**Published:** 2020-12-09

**Authors:** Haixiang Shen, Jin Liu, Guoliang Sun, Libin Yan, Qinchen Li, Zhize Wang, Liping Xie

**Affiliations:** 1 Department of Urology, First Affiliated Hospital, Zhejiang University School of Medicine, Hangzhou, Zhejiang Province, China; 2 Department of Surgical Oncology, First Affiliated Hospital, Zhejiang University School of Medicine, Hangzhou, Zhejiang Province, China; 3 Department of Urology, Tongji Hospital, Tongji Medical College, Huazhong University of Science and Technology, Wuhan, China

**Keywords:** PD-L1, prostate cancer, prognosis, clinicopathological, meta-analysis

## Abstract

Background: Programmed death-ligand 1 (PD-L1) is considered an adverse factor predicting poor prognosis in various cancers, but the significance of PD-L1 expression for the prognosis of prostate cancer (PCa) is still unclear. We aimed to investigate the clinicopathological significance and prognostic value of PD-L1 expression in PCa.

Methods: Studies were retrieved from PubMed, Web of Science, Cochrane Library and Embase before March 23, 2020. Odds ratios (ORs) and hazard ratios (HRs) with 95% confidence intervals (CIs) were obtained to assess the results. Begg’s test was applied to evaluate publication bias.

Results: Fourteen studies involving 3133 cases were analyzed. The pooled data showed that both PD-L1 protein expression and PD-L1 DNA methylation (mPD-L1) were negatively associated with biochemical recurrence-free survival, with HRs of 1.67 (95% CI = 1.38-2.06, *p* < 0.001) and 2.23 (95% CI = 1.51-3.29, *p* < 0.001), respectively. In addition, PD-L1 overexpression was significantly related to advanced tumor stage (OR = 1.40, 95% CI= 1.13-1.75, *p* = 0.003), positive surgical margin (OR = 1.36, 95% CI = 1.03-1.78, *p* = 0.028), higher Gleason score (OR = 1.81, 95% CI = 1.35-2.42, *p* < 0.001) and androgen receptor positivity (OR = 2.20, 95% CI = 1.61-3.01, *p* < 0.001), while no significant correlation with age (*p* = 0.122), preoperative PSA (*p* = 0.796) or nodal status (*p* = 0.113) was observed.

Conclusions: The study revealed that high expression of PD-L1 was related to unfavorable prognosis and advanced clinicopathological factors in PCa patients.

## INTRODUCTION

As the most common malignancy of the male urogenital system, prostate cancer (PCa) has become an increasingly serious threat to male patients. [1, 2]. In the United States, it was reported that PCa is one of the most common cancer diagnosed in men, with 1,746,50 new cases and 31,620 deaths expected in 2019 [1]. PCa is the most frequently diagnosed cancer among the male population in more than half (105/185) of the countries globally and has become the major reason for tumor-related mortality among men in 46 countries [2]. Fortunately, the mortality rate of PCa has become stabilized, even decreasing recently, which is attributed to earlier diagnosis and advanced treatments [1, 3, 4]. The 5-year relative survival rate for all stages of PCa is approximately 98% [1], while for only patients with advanced tumor stage, it decreases to 28% [5].

Immunotherapy, as an important part of cancer treatment, has garnered increasing attention in recent years with further understanding of the immune escape mechanism of tumor cells [6, 7]. It has been demonstrated that the immune checkpoint signaling pathway constituted by PD-L1 and programmed cell death 1 (PD-1) promotes immune evasion of tumors [8–10]. Previous studies illustrated that high PD-L1 expression indicates poor prognosis in numerous cancers, including breast cancer [11], lung cancer [12], renal cell carcinoma [13], esophageal squamous cell carcinoma [14], biliary tract cancer [15] and gastric cancer [16]. By blocking the interaction of PD-L1 and PD-1 to prevent immune evasion of tumors, anti-PD-L1 therapy with monoclonal antibodies such as avelumab has been proven effective for cancers like Merkel cell carcinoma and non-small cell lung cancer [17, 18].

Several pilot studies have revealed PD-L1 expression features in PCa, while the clinicopathological characteristics and the prognostic value of PD-L1 still remains unclear. Therefore, we conducted this study to determine whether PD-L1 affects the prognosis of PCa. In addition, we also explored the correlation between PD-L1 and clinicopathological factors.

## RESULTS

### Search results

**Figure 1:**
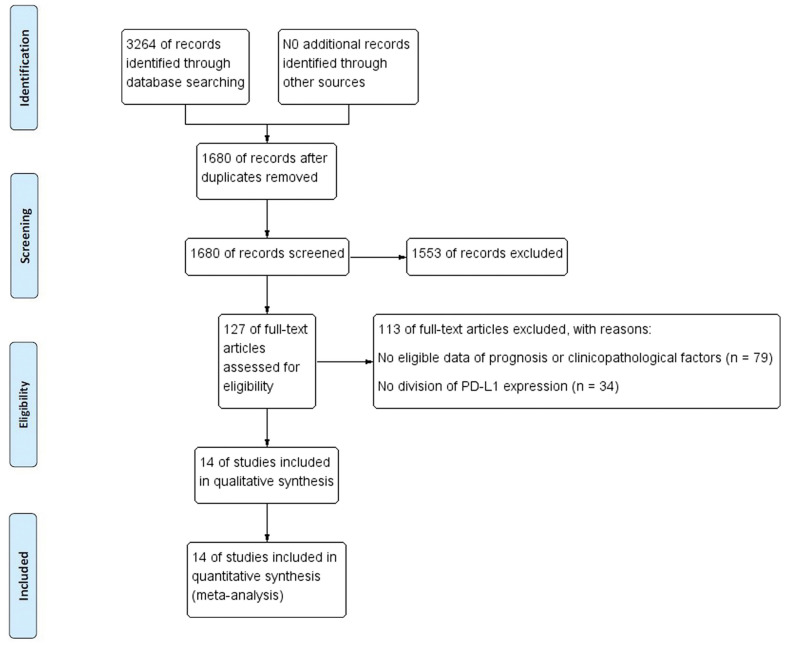
**Flow chart of study selection process.**

The literature retrieval process is displayed in Figure 1. A total of 3264 studies were acquired from the initial search. After duplicates were removed, 1680 articles were screened. After removal according to titles and abstracts, 127 studies remained. When assessing the full text, 79 articles were excluded for lacking data on prognosis or clinicopathological characteristics, and 34 studies were excluded for not distinguishing high and low PD-L1 expression. Ultimately, a total of 14 studies with 3133 patients published from 2009 to 2019 were included in the final analysis [19–23, 24–32]. Table 1 shows the baseline characteristics of all included studies. The assays used to detect PD-L1, evaluation methods and cutoff values are summarized in Supplementary Table 1. Among the final studies, 5 studies were included in the analysis of the effect of PD-L1 expression or PD-L1 DNA methylation (mPD-L1) on BCR-FS, and studies ranged from 1 to 10 in the analysis of the relationship of PD-L1 and various clinicopathologic factors. The quality of all qualified studies was high with the NOS scores ranging from 6 to 7. More details are displayed in Tables 2, 3.

**Table 1:** Characteristics of studies included in the meta-analysis.

**Study**	**Country**	**PD-L1 detection assay**	**PD-L1 positive (%)**	**Patient characteristics**								
				**Tumor status**	**Patients, n**	**Median age, yr (range)**	**Gleason score, n (%)**	**Tumor stage, n (%)**	**Surgical margin, n (%)**	**Nodal status, n (%)**	**Median PSA, ng/ml (range)**	**Median follow-up, month (range)**
Ness 2017	Norway	IHC	236/402 (58.7)	PCa following RP	535	62(47-75)	>8, 35(6.5) ≤8, 500(93.5)	pT1/pT2, 374(69.9) pT3/pT4, 161(30.1)	Positive, 286(53.5) Negative, 249(46.5)	NA	PSA<10, 308(57.6) PSA>10, 221(41.3) U, 6(1.1)	150(17-245)
Massari 2016	Italy	IHC	8/16 (50.0)	CRPC	16	64(53-70)	>8, 10(62.5) ≤8, 6(37.5)	NA	NA	NA	NA	at least 5 years
Calagua 2017	USA	IHC	18/130 (13.8)	hormone-naive primary cancer and prostate cancer underwent RP after Neo-AAPL	130	61(NA)^‡^	≥8, 34(26.0) ≤8, 96(74.0)	pT1/pT2, 64(49.2) pT3/pT4, 66(50.8)	Positive, 40(30.8) Negative, 90(69.2)	pN0, 98(75.4) pN1, 8(6.1) U, 24(18.5)	6(4.4, 9.1)	NA
Baas 2017	USA	IHC	2/25 (8.0)	high-grade Gleason 8-10 cancer	25	64±7.2 (50-79)^§^	>8, 8(32.0) ≤8, 17(68.0)	NA	NA	NA	13.9±14.3(2.4-68.9)^§^	NA
Fankhauser 2018-localized prostate cancer	Switzerland	IHC	0/96 (0.0)	localized prostate cancer	96	NA	NA	NA	NA	NA	NA	NA
Fankhauser 2018-CRPC	Switzerland	IHC	5/81 (6.2)	CRPC	81	75(54-86)	NA	NA	NA	NA	NA	NA
Haffner 2018-Primary Tumors	USA	IHC	39/508 (7.7)	primary cancer	508	NA	≥8, 111(21.9) ≤8, 397(78.1)	T1/T2, 195(38.4) T3/T4, 297(58.5) U, 16(3.1)	NA	N0, 467(91.9) N1, 36(7.1) U, 5(1)	NA	NA
Haffner 2018-CRPC	USA	IHC	18/57 (31.6)	metastatic CRPC	57	NA	NA	NA	NA	NA	NA	NA
Petitprez 2018	Italy	IHC	7/51 (13.7)	node-positive PCa treated with RP and ePLND	51	65(60-72)	>8, 17(33.0) ≤8, 34(67.0)	pT1/pT2, 8(16) pT3/pT4, 43(84)	Positive, 22(43) Negative, 29(57)	pN0, 50(98) pN1, 1(2)	9.9(6.6–15.3)	51(30–77)^¶^
Ebelt 2009-PCa	Germany	IHC	3/17 (17.6)	PCa following RP	17	66(59–75)	>8, 1(5.9) ≤8, 16(94.1)	pT1/pT2, 11(64.7) pT3/pT4, 6(35.3)	NA	NA	NA	NA
Gevensleben 2016a-PD-L1 training cohort	Germany	IHC	109/209 (52.2)	PCa following RP	209	65(45-83)	>8, 14(6.7) ≤8, 190(90.9) U, 5(2.4)	pT1/pT2, 124(59.3) pT3/pT4, 85(40.7)	Positive, 83(39.7) Negative, 124(59.3) U, 2(1.0)	pN0, 192(91.9) pN1, 16(7.7) U, 1(0.5)	7.5(0.7-163)	61.0(0-140)
Gevensleben 2016a-PD-L1 test cohort	Germany	IHC	377/611 (61.7)	PCa following RP	611	62(43-74)	>8, 40(6.5) ≤8, 571(93.5)	pT1/pT2, 418(68.4) pT3/pT4, 193(31.6)	Positive, 169(27.7) Negative, 439(71.8) U, 3(0.5)	pN0, 299(48.9) pN1, 9(1.5) U, 303(49.6)	7.1(0.8-39.0)	49.6(0-129)
Gevensleben 2016a-mPD-L1 training cohort	Germany	qPCR	101/498 (20.3)	PCa following RP	498	61(NA)	>8, 141(28.3) ≤8, 357(71.7)	pT2, 188(37.8) pT3, 303(60.8) U, 7(1.4)	Positive, 152(30.5) Negative, 316(63.5) U, 30(6.0)	pN0, 346(69.5) pN1, 79(15.9) U, 73(14.7)	PSA≤10, 339(68.0) PSA>10, 156(31.3) U, 3(0.6)	16(1-133)
Gevensleben 2016b-mPD-L1 validation cohort	Germany	qPCR	102/299 (34.1)	PCa following RP	299	NA	>8, 16(5.4) ≤8, 281(93.9) U, 2(0.7)	pT2, 205(68.6) pT3, 94(31.4) U, 1(0.3)	Positive, 98(32.8) Negative, 197(65.9) U, 4(1.3)	pN0, 278(93.0) pN1, 20(6.7) U, 1(0.3)	PSA≤10, 200(66.9) PSA>10, 86(28.8) U, 13(4.3)	63(1-145)
Iacovelli-2019-mCSPC	Italy	IHC	15/32 (46.9)	mCSPC	32	71.4(NA)	≥8, 29(90.6) <8, 3(9.4)	NA	NA	N0, 17(91.9) N1, 15(7.1)	170.0(NA)	83.4(NA)
Li 2019	China	IHC	63/127 (49.6)	high risk PCa received AHT after RP	127	66(48-76)	≥8, 63(50.4) <8, 64(49.6)	pT1/pT2, 54(42.5) pT3/pT4, 73(57.5)	Positive, 47(63.0) Negative, 80(37.0)	pN0, 86(67.7) pN1, 41(32.3)	49.74(1.98-408.21)	40(29-53)^¶^
Sharma 2019	USA	IHC	29/220 (13.2)	PCa following RP	220	60.3(42-78)	≥8, 24(10.9) <8, 196(89.1)	pT1/pT2, 166(75.5) pT3/pT4, 54(24.5)	NA	pN0, 138(62.7) pN1, 11(5.0) pNX, 71(32.3)	NA	48.2(3-116)
Xian 2019	USA	IHC	50/279 (17.9)	PCa following RP	279	61.1(39-76)	≥8, 73(26.2) <8, 206(73.8)	T1/T2, 168(69.2) T3/T4, 111(39.8)	NA	N0, 255(91.4) N1, 21(7.5) U, 3(1.0)	≤10, 220(78.9) >10, 54(19.4) U, 5(1.8)	106.5(3-180)

PSA, prostate specific antigen; IHC, immunohistochemistry; PCa, prostate cancer; pT, pathological tumor stage; pN, nodal pathological status; U, unknown; NA, not available; CRPC, castration-resistant prostate cancer; RP, radical prostatectomy; Neo-AAPL, neoadjuvant abiraterone acetate plus leuprolide plus prednisone; mCSPC, metastatic castration-sensitive prostate cancer; qPCR, quantitative polymerase chain reaction.

^‡^ Median age at diagnosis

^§^ Mean ± Standard deviation (Range)

^¶^ Median (Interquartile range)

**Table 2:** Newcastle-Ottawa scale for risk of bias assessment of the case control studies.

**Source**	**Selection**				**Comparability**	**Exposure**			
**Study**	**Adequacy of case definition**	**Representativeness of the cases**	**Selection of Controls**	**Definition of Controls**	Comparability of cases and controls on the basis of the design or analysis	**Ascertainment of exposure**	**Same method of ascertainment for cases and controls**	**Non-Response rate**	**Overall**
**Calagua 2017**	★	★			★★	★	★	★	7
**Haffner 2018**	★	★			★★	★	★	★	7
**Ebelt 2009**	★	★			★	★	★	★	6
**Baas 2017**	★	★			★★	★	★	★	7
**Fankhauser 2018**	★	★			★	★	★	★	6

**Table 3:** Newcastle-Ottawa scale for risk of bias assessment of the cohort studies.

**Source**	**Selection**			**Comparability**	**Outcome**			
**Study**	**Representativeness of exposed cohort**	**Selection of non-exposed cohort**	**Ascertainment of exposure to implants**	**Outcome not present at start**	Comparability of cohorts on the basis of the design or analysis	**Assessment of outcome**	**Adequate follow-up length**	**Adequacy of follow-up**	**Overall**
**Gevensleben 2016a**	★		★		★★	★	★	★	7
**Gevensleben 2016b**	★		★		★★	★	★	★	7
**Massari 2016**	★		★		★	★	★	★	6
**Ness 2017**	★		★		★★	★	★		6
**Petitprez 2017**	★		★		★★	★	★	★	7
**Iacovelli 2019**	★		★		★	★	★	★	6
**Li 2019**	★		★		★★	★	★	★	7
**Sharma 2019**	★		★		★★	★	★	★	7
**Xian 2019**	★		★		★★	★	★	★	7

### Prognostic significance of PD-L1 and mPD-L1 in PCa

**Figure 2:**
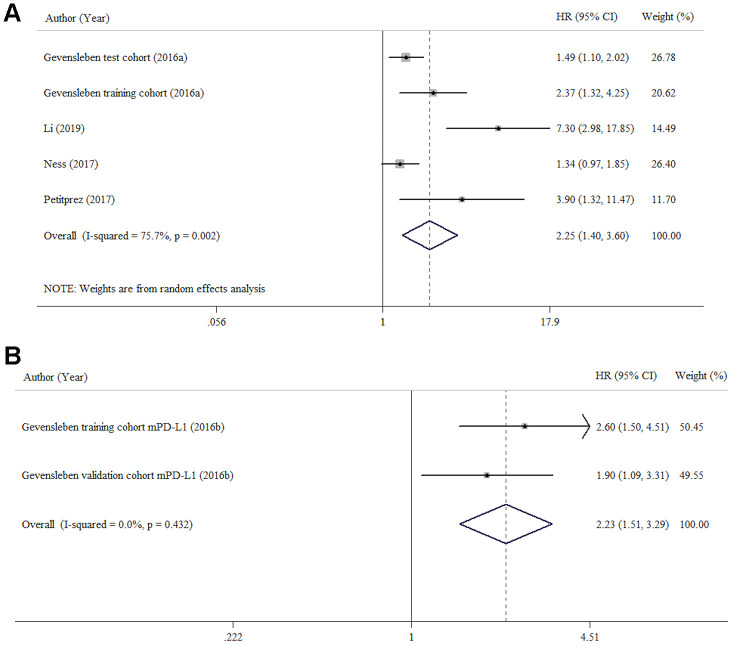
****Forest plots HR for assessing the association between BCR-FS and PD-L1 protein expression (**A**) and mPD-L1 (**B**) in patients with prostate cancer.

Five studies [19–23] reported information about univariate proportional hazards analysis of PD-L1 expression or mPD-L1. The pooled results are displayed in Figure 2A and Table 4 (HR = 1.67, 95% CI = 1.38-2.06, *p* < 0.001), demonstrating that PD-L1 overexpression predicted poor BCR-FS. However, high heterogeneity was detected among the studies (I^2^ = 75.7%, *p* = 0.002). As seen in Figure 2B, we identified a significant association between high mPD-L1 and poor BCR-FS (HR = 2.23, 95% CI, 1.51-3.29, *p* < 0.001). No significant heterogeneity was present (I^2^ = 0.0%, *p* = 0.430).

**Table 4:** Meta-analysis results for the clinicopathological significance and prognostic value of PD-L1 in PCa.

	**No. of studies**	**Chi²**	***p*_heterogeneity_**	**I² (%)**	**Pooled OR/HR (95% CI)**				**Begg's test**
					**Fixed model**	***p* value**	**Random model**	***p* value**	***p* value**
Age (>60 VS ≤60)	3	0.37	0.947	0.0	1.28 (0.94-1.75)	0.122	1.28 (0.94-1.75)	0.123	0.734
Preoperative PSA (>10 VS ≤10)	2	4.92	0.085	59.3	1.04 (0.78-1.39)	0.796	0.91 (0.54-1.55)	0.733	1.000
Tumor stage (pT3,4 VS pT1,2)	7	10.87	0.144	35.6	1.40 (1.13-1.75)	0.003	1.46 (1.08-1.99)	0.015	0.386
pN (N1 VS N0)	7	15.96	0.025	56.1	1.37 (0.93-2.03)	0.113	1.53 (0.80-2.93)	0.199	0.108
Surgical margin (R1 VS R0)	3	5.33	0.149	43.8	1.36 (1.03-1.78)	0.028	1.49 (0.99-2.23)	0.055	0.308
Gleason score (>8 VS ≤8)	10	12.47	0.255	19.8	1.81 (1.35-2.42)	<0.001	1.87 (1.32-2.66)	<0.001	0.436
AR status (AR+ VS AR-)	1	1.1	0.294	9.3	2.20 (1.61-3.01)	<0.001	2.22 (1.58-3.10)	<0.001	1.000
PD-L1 expression (CRPC VS HSPC)	2	0.42	0.515	0.0	6.01 (3.22-11.23)	<0.001	5.64 (3.04-10.49)	<0.001	1.000
BCR-FS (PD-L1 high VS PD-L1 low)	5	16.49	0.002	75.7	1.67 (1.38-2.06)	<0.001	2.25 (1.40-3.61)	0.001	0.221
BCR-FS (mPD-L1 high VS low)	1	0.52	0.432	0.0	2.23 (1.51-3.29)	<0.001	2.23 (1.51-3.29)	<0.001	1.000

### PD-L1 expression in castration-resistant prostate cancer (CRPC) and primary PCa

**Figure 3:**
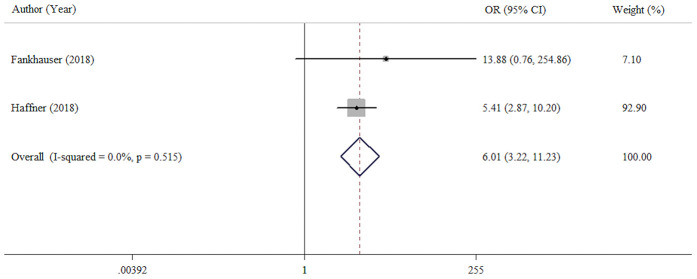
**Forest plot assessing the association between PD-L1 expression and sensitivity of androgen deprivation therapy (CRPC VS HSPC).**

We explored the association of PD-L1 expression with sensitivity to androgen deprivation therapy (CRPC vs primary PCa). This analysis included two studies with 819 patients. Compared with primary PCa cases (hormone-sensitive prostate cancer, HSPC), CRPC cases had a high prevalence of high or positive PD-L1 expression (OR = 6.01, 95% CI = 3.22-11.23, *p* < 0.001) (Figure 3). No significant heterogeneity was found (I^2^ =0.0%, *p* = 0.520).

### The clinicopathological significance of PD-L1 expression in PCa

**Figure 4:**
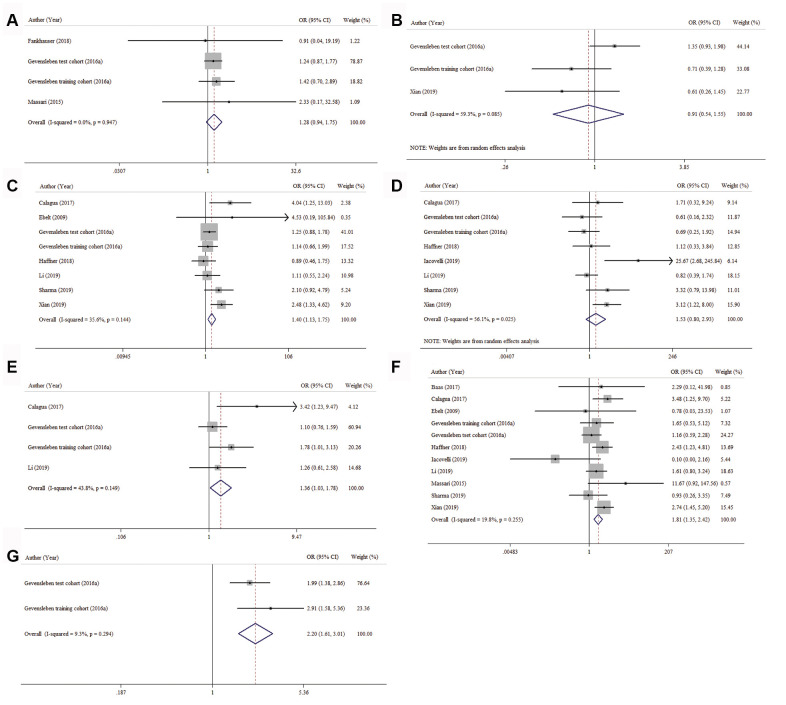
**Forest plots for the correlation between PD-L1 expression and clinicopathologic characteristics.** (**A**) age, (**B**) preoperative PSA, (**C**) tumor stage, (**D**) nodal status, (**E**) surgical margin, (**F**) Gleason score and (**G**) AR status.

To find out the association between PD-L1 expression and age, preoperative PSA, tumor stage, nodal status, surgical margin, Gleason score and AR status, 3, 2, 7, 7, 3, 10 and 1 studies were analyzed, respectively. As shown in Figure 4A–4G and Table 4, the pooled ORs revealed that PD-L1 overexpression was more prevalent in PCa patients with advanced tumor stage (OR = 1.40, 95% CI = 1.13-1.75, *p* = 0.003), positive surgical margin (OR = 1.36, 95% CI = 1.03-1.78, *p* = 0.028), higher Gleason score (OR = 1.81, 95% CI = 1.35-2.42, *p* < 0.001) and AR positivity (OR = 2.20, 95% CI = 1.61-3.01, *p* < 0.001). However, the associations between PD-L1 expression and age, preoperative PSA and nodal status were not statistically significant (*p* = 0.122, *p* = 0.796, and *p* = 0.113, respectively).

### Publication bias assessment

Begg’s test was applied to assess publication bias. The results suggested that no evidence of significant publication bias was present (Table 4 and Supplementary Figure 1A–1I).

## DISCUSSION

PD-L1 is a type 1 transmembrane protein, a member of the B7/CD28 costimulatory factor family, which was first discovered by Dong in 1999 [33]. PD-L1 is normally expressed by macrophage lineage cells with the induction of inflammatory cytokines [33–35]. PD-L1 can function as an immune checkpoint. When PD-L1 binds to its receptor PD-1, the immune system is negatively regulated to protect tissues from damage in normal physiological situations [9, 36]. However, PD-L1 is also expressed in numerous tumor cells [37–41]. Overexpression of PD-L1 in tumor cells combined with PD-1, which is mainly expressed on activated T-cells, can trigger an immunosuppression effect in the tumor microenvironment, leading to tumor immune evasion [9, 10]. It was reported that PD-L1 positivity on tumor cells of primary PCa and CRPC is ~92% and ~19%, respectively [20, 21, 26]. And the expression of PD-L1in PCa patients will up-regulate in response to inflammatory cytokines like IFN-γ or when particular signaling pathways (NF-kB) is activated [42].

Recently, some studies have verified that PD-L1 overexpression indicates poor prognosis in various cancers [15, 43–46], and several anti-PD-L1 monoclonal antibodies, including avelumab, durvalumab and atezolizumab have been approved by the FDA [47–49]. However, anti-PD-L1 therapy in PCa is not as effective as it is in other solid tumors. Few studies have explored the prognostic value of PD-L1 in PCa with disputable and unclear results.

This meta-analysis was performed to explore whether the prognosis of PCa correlates with PD-L1. The pooled data from the eligible studies revealed that overexpressed PD-L1 and mPD-L1 predicted poor BCR-FS in PCa patients, which was consistent with Li’s study [50].

Our study also explored the relationship of PD-L1 expression with clinicopathological features in PCa. The pooled results revealed that high PD-L1 expression was more likely to be observed in patients with CRPC than in patients with HSPC. This finding indicated that patients with PD-L1 overexpression show more resistance to androgen deprivation therapy (ADT) than those with low PD-L1 expression and might obtain a survival benefit from anti-PD-L1 immunotherapy. Additionally, PD-L1 overexpression was more prevalent in patients with advanced tumor stage, high GS, and positive surgical margin and positive AR status, which suggested the potential of a combined strategy featuring anti-PD-L1 immunotherapy and ADT or radiotherapy in consideration in certain situations.

Li [50] reported no statistically significant association between PD-L1 and age (OR = 1.27, 95% CI = 0.93-1.75, *p* = 0.14), nodal status (OR = 0.65, 95% CI = 0.35-1.21, *p* = 0.17) or preoperative PSA (OR = 1.13, 95% CI = 0.82-1.54, *p* = 0.46). A study conducted immunohistochemical staining of PD-L1/PD1 in 279 patients who underwent radical prostatectomy indicated that age (≥ 65 years), obesity (BMI ≥ 30), and advanced tumor stage, lymph node metastasis, and high Gleason score patients were related with higher PD-L1 positivity [32]. In total, 6.5 tumor-infiltrating lymphocytes per high power field were positive for PD-1 staining and 50/279 (17.9%) tumors were positive for PD-L1 staining. Interestingly, Peng’s research indicated PD-1-positive lymphocytes were significantly more expressed in PD-L1-positive tumors than PD-L1-negative.

Similarly, the correlation of PD-L1 over-expression with advanced tumor stage and higher Gleason score were also found in our study, but for age, preoperative PSA or nodal status was not significant. Moreover, we indicated that surgical margin and androgen receptor was relevant to higher PD-L1 expression. Furthermore, our study demonstrated that the prevalence of PD-L1 overexpression was higher in pT3/pT4 stage than other stages in PCa (OR = 1.40, 95% CI = 1.13-1.75, *p* = 0.003).

We conducted this study to uncover the prognostic and clinicopathological value of PD-L1 in PCa. Compared with previous research, we included higher numbers of qualified studies and performed more comprehensive analyses. Furthermore, this study was the first to uncover the obvious correlation between PD-L1 expression and surgical margin. However, there are several limitations in our study that should be stated. First, even though the same detection assay, immunohistochemistry, was applied to detect the expression of PD-L1, the specifics of the assays varied between studies. Second, the evaluation methods and cutoff values for dichotomization were inconsistent between eligible studies. Both of the above factors could be the origins of heterogeneity. Third, due to a lack of eligible data, subgroup analyses for BCR-FS based on tumor stage and various therapies were not be performed. Fourth, this meta-analysis included relatively few studies, and the total case number was not large enough. Fifth, most of the eligible studies included were retrospective in design.

## CONCLUSIONS

In summary, our results clearly revealed that PD-L1 overexpression and mPD-L1 had value for predicting poor BCR-FS in PCa. Furthermore, the study also uncovered a significant correlation between PD-L1 overexpression and the clinicopathological features of CRPC: advanced tumor stage, higher Gleason score, positive surgical margin and positive AR status. These findings could be helpful for clinical decision making. Nevertheless, more multicenter prospective studies with large sample sizes, long observation periods and well-designed methods are required to draw a more reliable conclusion.

## MATERIALS AND METHODS

This meta-analysis was conducted according to Preferred Reporting Items for Systematic Reviews and Meta-Analyses (PRISMA) principles [51].

### Literature search

The retrieve was conducted on PubMed, Cochrane Library, Web of Science and Embase to identify relevant articles published prior to March 23, 2020. The free text terms and medical subject headings (MeSH) terms used in search covered “prostate cancer” OR “prostate tumor” OR “prostate neoplasm” OR “prostate carcinoma”; “programmed death-ligand 1” OR “B7-H1” OR “programmed cell death-ligand 1” OR “CD274” OR “PD-L1”.

### Inclusion and exclusion criteria

Two researchers (HS and JL) identified the titles, abstracts, and whole articles independently, with disagreements settled by discussion. The inclusion criteria were: (1) studies were published in English; (2) PCa was confirmed by histopathological examination; (3) PD-L1 protein or mPD-L1 was evaluated in PCa tissues; (4) the expression level of PD-L1 was assessed with positive (high) or negative (low) labels; (5) studies reported the relationship of PD-L1 and clinicopathological characteristics or prognosis. The exclusion criteria were: (1) studies only reported animals or *in vitro* experiments; (2) duplicate studies; (3) reviews, meta-analyses, meeting abstracts, expert opinions, letters, editorials, or case reports.

### Data extraction

Data was collected by two reviewers (HS and JL) independently and differences were resolved by discussion. The data gathered were as follows: author name, publication year, country, size of the study population, age, preoperative PSA, Gleason score, tumor stage, nodal status, surgical margin, the expression level of PD-L1, hazard ratio (HR) and 95% confidence interval (CI) for BCR-FS, follow-up period, detection assay, evaluation method and cut-off value.

The Newcastle-Ottawa Scale (NOS) containing three domains: (1) selection; (2) comparability; (3) exposure or outcome, was implemented to evaluate the study quality [52]. High quality was considered when the score of NOS was more than 5.

### Statistical analysis

Pooled HRs was calculated with 95% CIs to evaluate the PD-L1 value in prognosis of PCa patient. The odd ratios (ORs) with 95% CIs was computed to reveal the correlation between PD-L1 and clinicopathological factors. Patients were divided into two groups by age (>60 VS ≤60), preoperative PSA (>10 VS ≤10), surgical margin (R1 vs R0), tumor stage (pT3-T4 vs pT1-T2), nodal status (pN1 vs pN0), Gleason score (>8 vs ≤8) and androgen receptor (AR) expression (positive vs negative). Statistical heterogeneity of different studies was determined via employing the Chi-square-based Q statistics and I^2^ value [53]. If I^2^ > 50% and *p* < 0.1, the heterogeneity was considered to be high, then the random-effects model was implemented. If not, a fixed-effects model was applied.

We used Begg’s test to estimate the potential publication bias of included studies. All the statistical analyses were realized by STATA software (version 12.0, Stata Corp LP, TX77845, USA). Two-tailed *p*-value < 0.05 was regarded statistically significant.

## Supplementary Material

Supplementary Figure 1

Supplementary Table 1
