# Supplementary material for: The clinicopathological significance and prognostic value of programmed death-ligand 1 in prostate cancer: a meta-analysis of 3133 patients
Source: Aging (Albany NY). 2020 Dec 9;13(2):2279–93. doi: 10.18632/aging.202248 (PMC7880326; doi:10.18632/aging.202248)
Supplement: Supplementary Figure 1 [file aging-13-202248-s001.pdf]

SUPPLEMENTARY FIGURE

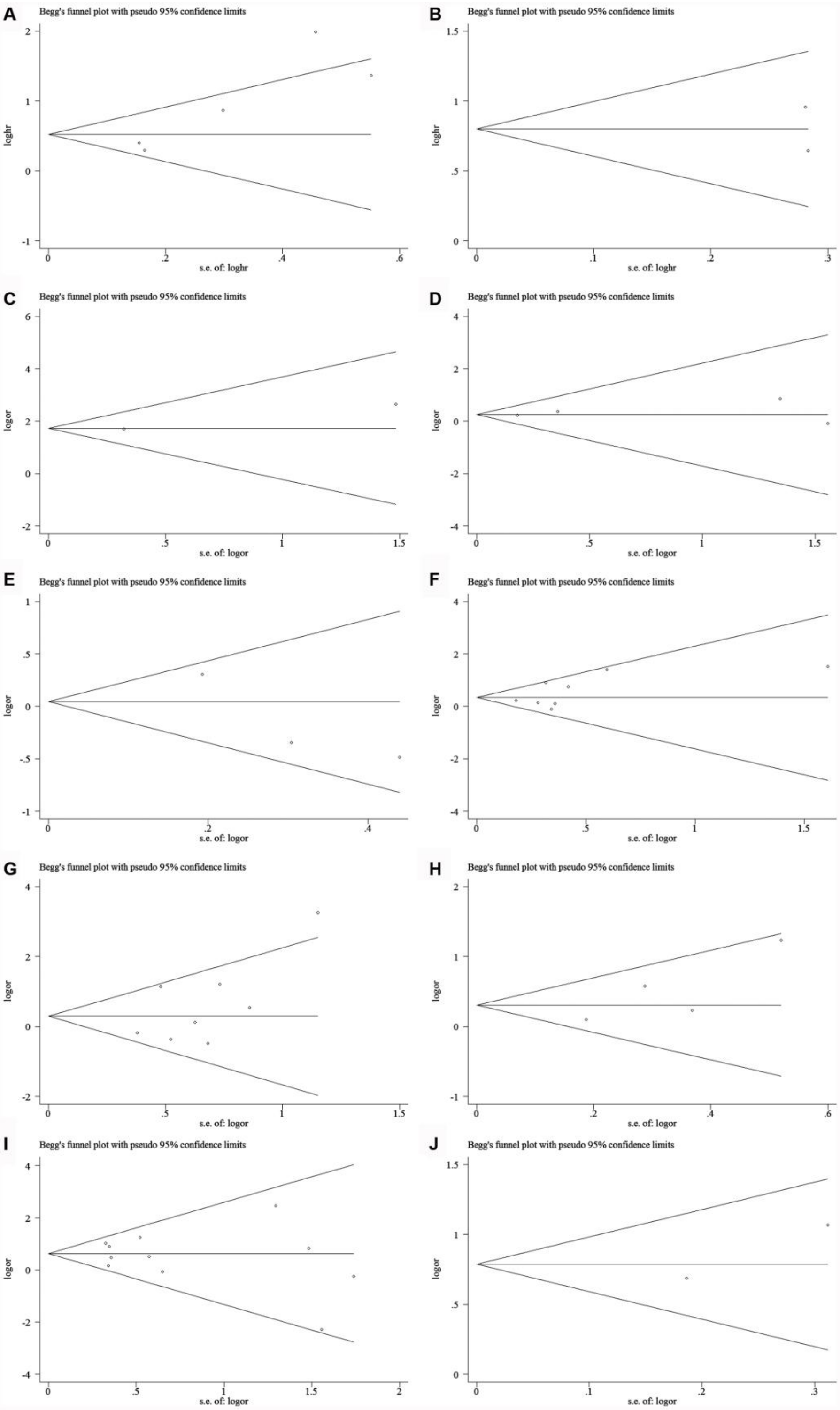

Supplementary Figure 1. The Begg's funnel plots for publication bias of (**A**) BCR-FS with PD-L1, (**B**) BCR-FS with mPD-L1, (**C**) PD-L1-positive proportion, (**D**) age, (**E**) pre-operative PSA, (**F**) tumor stage, (**G**) nodal status, (**H**) surgical margin, (**I**) Gleason score and (**J**) AR status.
