# Supplementary material for: The clinicopathological significance and prognostic value of programmed death-ligand 1 in prostate cancer: a meta-analysis of 3133 patients
Source: Aging (Albany NY). 2020 Dec 9;13(2):2279–93. doi: 10.18632/aging.202248 (PMC7880326; doi:10.18632/aging.202248)
Supplement: Supplementary Table 1 [file aging-13-202248-s002.docx]

**Supplement Table 1 – Target specimens, assays, dichotomization forms,** **evaluation target cells, evaluation methods and cut-off values of PD-L1 detection in the meta-analysis.**

| Study | Target specimens | PD-L1 detection assay | Dichotomization form  (PD-L1-positive / negative status or PD-L1 high / low expression) | Evaluation target cells | Evaluation methods and cut-off values |
| --- | --- | --- | --- | --- | --- |
| Ness 2017 | TMA from FFPE prostatectomy tissue | Immunohistochemistry  It was performed on a Discovery-Ultra immunostainer (Ventana Medical Systems, Tucson, AZ) using antibody PD-L1 with 1/25 dilution (Cat#13684, clone: E1L3N, Cell Signaling Technology, Danvers, MA, USA). | PD-L1 high / low expression | Tumor epithelial cells and tumor stromal cells | An intensity scoring scale was chosen: no staining = 0, weak staining=1, moderate staining=2, and strong staining=3.  Cut-off values for dichotomization of low and high expression were chosen according to a minimal P-value approach (optimal cut-off) and high scores were defined as follows: (i)  ≥ 0.54 (mean) for PD-L1+ tumor stromal cells (ii) ≥ 1.0 for PD-L1 tumor epithelial cells. |
| Massari 2016 | TMA from FFPE prostatectomy tissue | Immunohistochemistry  It was processed by a Bond Polymer Refine Detection system in an automated Bond immunostainer (Vision Biosystem, Menarini, Florence, Italy) using antibody; PD-L1 (clone 015, Sino Biological). | PD-L1-positive / negative status | Neoplastic cells | PD-L1 staining intensity was defined as follows: 0 (no signal), 1+ (light signal) and 2+ (high signal) in >50 % neoplastic cells. |
| Calagua 2017 | FFPE prostatectomy tissue | Immunohistochemistry  Dual immunostains for PD-L1 (E1L3N, Cell Signaling; 1:100) and CK18 (DC10, Dako; 1:4) were stained using Dako EnVision G\|2 Doublestain System following manufacturer’s protocol. | PD-L1-positive / negative status | Tumor cells | PD-L1 positivity was defined by moderate to strong membranous staining and cytoplasmic staining was not considered.  PD-L1 percentage scoring was performed semi-quantitatively as follows: 0 (negative or < 1%), 1 (1 - 4%), 2 (5 - 24%), 3 (25 - 49%), and 4 (≥ 50%).  Negative is defined as “< 1%”.  Here, we defined PD-L1 high expression as “≥ 50%”. |
| Baas 2017 | FFPE prostatectomy/biopsy tissue | Immunohistochemistry  It was performed at Merck Research Laboratories (Palo Alto, CA) using anti-PD-L1 (clone 22C3; Merck Research Laboratories). | PD-L1 high / low expression | Tumor cells | Samples were scored semi-quantitatively on a 0 to 5 scale. A score of 3 to 5 was deemed “high” expression whereas a score of 0 to 2 was deemed “low” expression. |
| Fankhauser 2018 - localized prostate cancer cohort | TMA from FFPE PCa tissue | Immunohistochemistry  It was performed by Ventana Benchmark automated staining system and Ventana reagents (both Ventana Medical Systems, Tucson, AZ, USA) using ONE anti-human PD-L1 rabbit monoclonal antibody: E1L3N (Cell Signaling Technology, Inc., Danvers, MA, USA). | PD-L1-positive / negative status | Tumor cells | Percentages of PD-L1 positive tumor cells and the staining pattern were evaluated and a > 1% cut-off for membranous PD-L1 positivity in tumor cells was used. |
| Fankhauser 2018 - CRPC cohort | TMA from FFPE CRPC tissue | Immunohistochemistry  It was performed by Ventana Benchmark automated staining system and Ventana reagents (both Ventana Medical Systems, Tucson, AZ, USA) using TWO anti-human PD-L1 rabbit monoclonal antibody: E1L3N (Cell Signaling Technology, Inc., Danvers, MA, USA) and SP263 (Ventana Medical Systems, Tucson, AZ, USA). | PD-L1-positive / negative status | Tumor cells | Percentages of PD-L1 positive tumor cells and the staining pattern were evaluated and a > 1% cut-off for membranous PD-L1 positivity in tumor cells was used. |
| Haffner 2018 – primary tumors | TMA from FFPE PCa tissue | Immunohistochemistry  It was performed by an automated instrument (BenchMark Ultra, Ventana medical systems, Inc. Tucson, AZ) using the rabbit monoclonal clone SP263, (Ref# 790-4905, Ventana Medical Systems, Inc. Tucson, AZ) | PD-L1-positive / negative status | Tumor cells | Any PD-L1-specific immunoreactivity on malignant cells (≥ 1%) was considered positive. |
| Haffner 2018- mCRPC | TMA from FFPE surgical resection or biopsy tissue | Immunohistochemistry  It was performed by an automated instrument (BenchMark Ultra, Ventana medical systems, Inc. Tucson, AZ) using the rabbit monoclonal clone SP263, (Ref# 790-4905, Ventana Medical Systems, Inc. Tucson, AZ) | PD-L1-positive / negative status | Tumor cells | Any PD-L1-specific immunoreactivity on malignant cells (≥ 1%) was considered positive. |
| Ebelt 2009 –primary tumors | Tissue samples from prostatectomy embedded in Tissue Tek® compound | Immunohistology  It was performed using anti-human B7-H1 (clone MIH1, BD Pharmingen) antibody | PD-L1-positive / negative status | Tumor cells and lymphocyte clusters | The number of B7-H1+ cells was evaluated semi-quantitatively in serial tissue cryosections by estimating the number of positively stained cells (in case of lymphocyte clusters) or by counting positive cells (in cases where positively stained cells were rare).  Tissues were grouped according to the estimated cell count as follows: “>1000”, “100-1000”, “50–100”, “10-50” or “<10”.  We defined “≥100” as PD-L1-positive. |
| Gevensleben 2016a – PD-L1 protein | TMA from FFPE prostatectomy tissue | Immunohistology  It was carried out on the Ventana BenchMark Ultra automated staining system (Ventana) and visualized with the Ventana amplifier detection kit using the following antibodies: PD-L1, clone EPR1161(2) (Abcam; 1:75) | PD-L1 high / low expression | Tumor cells | The staining was uniformly homogeneous, the intensity of PD-L1–positive cells was scored semi-quantitatively as negative (0), weak (1), mod- erate (2), or strong (3). PD- L1 expression dichotomized by median. |
| Gevensleben 2016b – mPD-L1 | FFPE prostatectomy tissue | PD-L1 quantitative methylation real-time PCR | mPD-L1 high / low | Tumor cells | mPD-L1_low_ < 0.98% ≤ mPD-L1_high_ |
| Petitprez 2017 | FFPE prostatectomy tissue | Immunohistology  It was performed using anti-human PD-L1 (mouse, clone 22C3, pharmDx, Dako) antibody | PD-L1-positive / negative status | Tumor cells | PD-L1_negative_ < 1% ≤ PD-L1_positive_ |
| Iacovelli 2019 - mCSPC | TMA from FFPE PCa tissue | Immunohistology  The PD-L1 IHC 28-8 pharmDx was used with monoclonal rabbit anti-PD-L1, clone 28-8. | PD-L1-positive / negative status | Tumor cells | 1% as a cut-off for PD-L1 expression |
| Li 2019 | FFPE prostatectomy tissue | Immunohistology  It was performed to assess protein expression of PDL1 (mouse monoclonal, ab210931, Abcam, Cambridge, UK, 1:200 dilution), PD1 (rabbit monoclonal, ab137132, Abcam, 1:200 dilution) by Bond Polymer Refine Detection System (Leica Biosystems Newcastle Ltd., Newcastle upon Tyne, UK). | PD-L1 high / low expression | Tumor cells | The intensity of PD-L1 positive cells was scored semi-quantitatively as negative (0), weak (1), moderate (2), or strong (3). A semi-quantitative score was implemented to evaluate each tissue. The percentage of stained cells (0–100%) was multiplied by the dominant intensity pattern of staining (0–3)  PD-L1 expression was dichotomized by median |
| Sharma 2019 | TMA from FFPE prostatectomy tissue | Immunohistology  It was performed using anti-human PD-L1 (IHC 22C3 pharmDx; Agilent Technologies) antibody | PD-L1-positive / negative status | Tumor cells/Tumor-infiltrating lymphocytes or macrophages | It was positive when moderate to strong staining was seen in at least 1% of tumor cells/tumor-infiltrating lymphocytes or macrophages, or weak staining was seen in at least 10% of tumor cells/tumor- infiltrating lymphocytes or macrophages. |
| Xian 2019 | FFPE prostatectomy tissue | Immunohistology  It was performed using rabbit monoclonal antibody against PD-L1 (Catalog# 13684, used at 1:240 dilution, Cell Signaling Technology, Danvers, MA, USA) | PD-L1-positive / negative status | Tumor cells | Any specimen with ≥ 1% positive tumor cells was considered as PD-L1 positive and any specimen with < 1% positive tumor cells was considered as PD-L1 negative. |

FFPE, formalin-fixed-paraffin-embedded; PCa, prostate cancer; TMA, Tissue microarrays; CRPC, castration-resistant prostate cancer; mPD-L1, PD-L1 promoter methylation, mCSPC, metastatic castration-sensitive prostate cancer
